# A novel crosstalk within the endocannabinoid system controls GABA transmission in the striatum

**DOI:** 10.1038/s41598-017-07519-8

**Published:** 2017-08-04

**Authors:** A. Musella, D. Fresegna, F. R. Rizzo, A. Gentile, S. Bullitta, F. De Vito, L. Guadalupi, D. Centonze, G. Mandolesi

**Affiliations:** 1 Centro Europeo per la Ricerca sul Cervello (CERC), IRCCS Fondazione Santa Lucia, 00143 Rome, Italy; 2 Unit of Neurology and of Neurorehabilitation, IRCCS Istituto Neurologico Mediterraneo (INM) Neuromed, 86077 Pozzilli (IS), Italy; 3 Department of Systems Medicine, Tor Vergata University, 00133 Rome, Italy

## Abstract

The N-palmitoylethanolamine (PEA) is an endogenous member of the endocannabinoid system (ECS) with several biological functions, including a neuromodulatory activity in the central nervous system. To shed light on the neuronal function of PEA, we investigated its involvement in the control of both excitatory and inhibitory transmission in the murine striatum, a brain region strongly modulated by the ECS. By means of electrophysiological recordings, we showed that PEA modulates inhibitory synaptic transmission, through activation of GPR55 receptors, promoting a transient increase of GABAergic spontaneous inhibitory postsynaptic current (sIPSC) frequency. The subsequently rundown effect on sIPSC frequency was secondary to the delayed stimulation of presynaptic cannabinoid CB1 receptors (CB1Rs) by the endocannabinoid 2-AG, whose synthesis was stimulated by PEA on postsynaptic neurons. Our results indicate that PEA, acting on GPR55, enhances GABA transmission in the striatum, and triggers a parallel synthesis of 2-AG at the postsynaptic site, that in turn acts in a retrograde manner to inhibit GABA release through the stimulation of presynaptic CB1Rs. This electrophysiological study identifies a previously unrecognized function of PEA and of GPR55, demonstrating that GABAergic transmission is under the control of this compound and revealing that PEA modulates the release of the endocannabinoid 2-AG.

## Introduction

In the last two decades, many physiological studies have substantially contributed to discover additional endogenous member of the endocannabinoid system (ECS) provided with distinct biological actions^1^. Among them, the endocannabinoid *N*-palmitoylethanolamine (PEA), was soon recognized as an important analgesic, anti-inflammatory and neuroprotective mediator, although its mechanism of action remains as yet elusive^2–4^.

Several research groups have proposed a neuromodulator role for PEA, based on the evidence that it is abundant in the central nervous system (CNS)^5, 6^, and is produced in discrete areas of the brain under both physiological and pathological conditions^7^.

PEA is an endogenous congener of the endocannabinoid anandamide (AEA) and is usually biosynthesized through similar metabolic pathways and enzymes together with AEA^8^ but, unlike AEA, PEA predominantly acts on G-protein coupled receptor, GPR55^9, 10^, showing low affinity for both type-1 and type-2 cannabinoid receptors (CB1 and CB2) and also for TRPV1 channels^3, 11^. Moreover, PEA has been involved in the activation of the nuclear receptor peroxisome proliferator activated receptor-α (PPAR-α) through which it plays a key role in the regulation of the inflammatory response and pain^11^.

GPR55 receptors have been heavily implicated in immunomodulatory functions but less is known about their effects on neuronal cells. These receptors are widely expressed in the brain and have been found significantly expressed in the striatum, although their cellular and subcellular localization is still unclear^12–15^. GPR55 stimulation was found to increase intracellular calcium in dorsal root ganglion neurons^16^, establishing this compound as a cannabinoid receptor with signaling distinct from CB1 and CB2 receptors. Of note, pharmacological modulation of GPR55 receptors has been reported to enhance the levels and/or actions of others endocannabinoids, at CB receptors and TRPV1 channels, a property known as the ‘entourage’ effect^17–20^. In line with this, Petrosino *et al*.^21^ showed that PEA exerts significant effect on endocannabinoid 2-arachidonoiylglicerol (2-AG) by enhancing its levels.

To shed light on the neuronal function of PEA, here we have investigated the involvement of this compound in the control of spontaneous synaptic transmission in striatal neurons. We found that PEA selectively modulates the frequency of GABA-mediated spontaneous currents (sIPSCs) through activation of GPR55 receptors. Our results also show that PEA, by acting on the same receptors, stimulates synthesis and delayed synaptic effects of the other endocannabinoid 2-AG, giving rise to a previously unrecognized feed-back control of GABA transmission mediated by endocannabinoids.

## Results

### Effect of PEA on striatal GABAergic synaptic transmission

**Figure 1:**
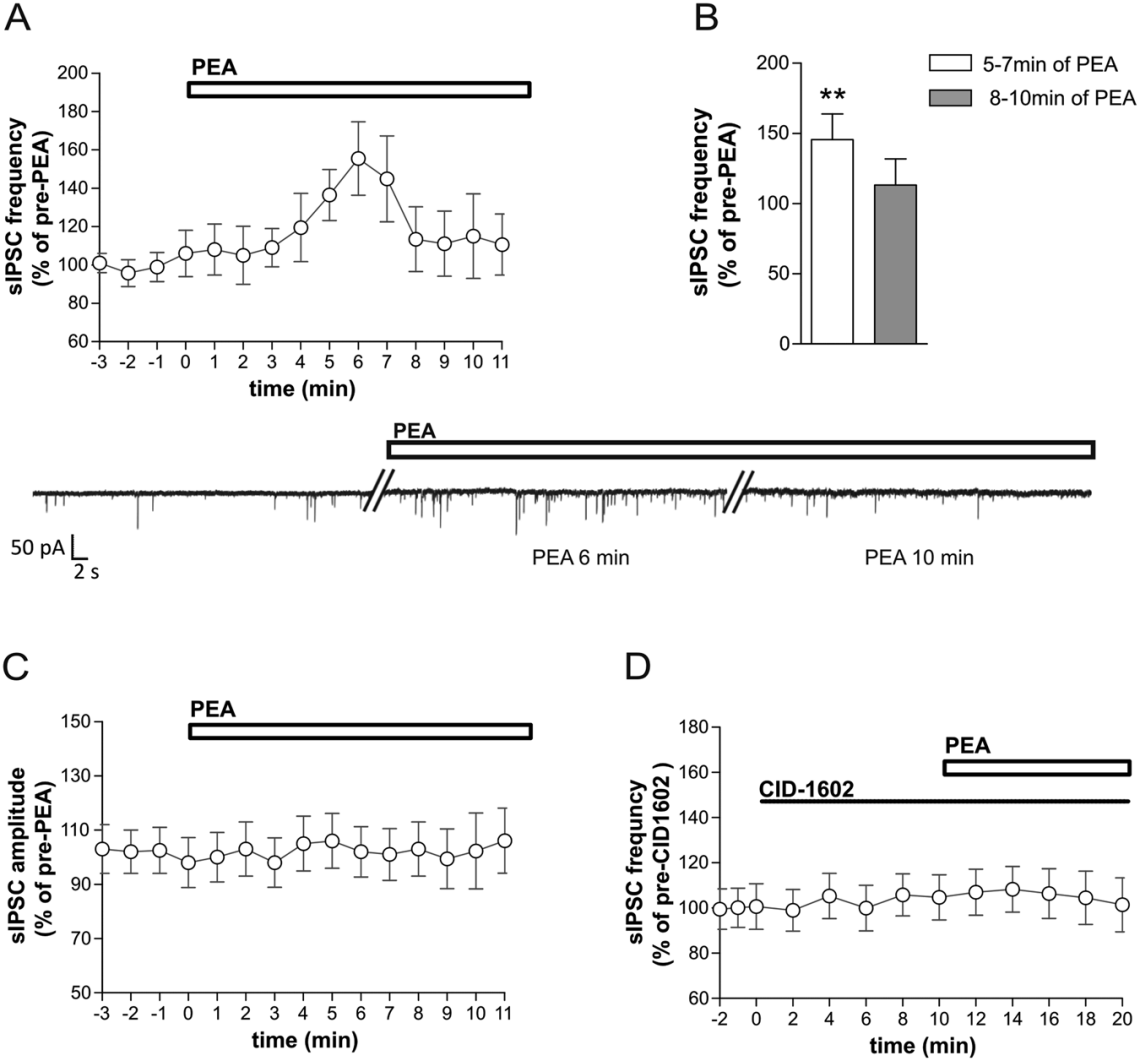
N-Palmitoylethanolamide (PEA) increases striatal GABA transmission. (**A,B**) The graphs show the early increase and the late reduction of sIPSC frequency after the application of PEA. The electrophysiological traces on the bottom are examples of voltage-clamp recordings before and during the application of PEA. (**C**) PEA-induced modifications of sIPSC frequency were not associated to any change in mean amplitude of sIPSCs. (**D**) The effects of PEA on sIPSC frequency were fully prevented by the application of CID-O1602. Paired Student’s t-Test **, p < 0.01.

Electrophysiological recordings were performed from medium spiny neurons to explore whether PEA was able to affect synaptic transmission in the striatum. Application of PEA caused a transient enhancement of GABA signaling, by increasing the frequency of spontaneous inhibitory postsynaptic currents (sIPSCs) without altering their amplitude. sIPSC frequency-time histogram indicated that the effect of PEA was maximal 5–7 min after its application (124 ± 11%, n = 8, p < 0.01 compared to pre-drug values), and underwent a rapid rundown during prolonged applications (106 ± 10%, n = 8 p > 0.05 compared to pre-drug values at 8–10 min) (Fig. 1 A and B). A presynaptic action likely mediated this effect since amplitude of sIPSCs was unchanged during the response to PEA (n = 8, p > 0.05 at all time points) (Fig. 1C).

Since it was reported that PEA acts as a GPR55 receptor agonist^9, 10^ and the GPR55 receptor was localized in the striatum of both humans and mice^14, 15^, we further characterized PEA-mediated synaptic alteration by performing electrophysiological recordings in the presence of a GPR55 antagonist, CID-1602. The increase of sIPSC frequency mediated by PEA was fully prevented in the presence of a GPR55 receptor antagonist/inverse agonist, CID-1602 (n = 6, p > 0.05) (Fig. 1D), indicating that the inhibitory synaptic currents (sIPSCs) were likely modulated by GPR55 receptors. Moreover, CID-1602 failed to alter sIPSCs when applied alone, suggesting that, in the striatum, GPR55 receptors are not tonically activated in basal condition.

**Figure 2:**
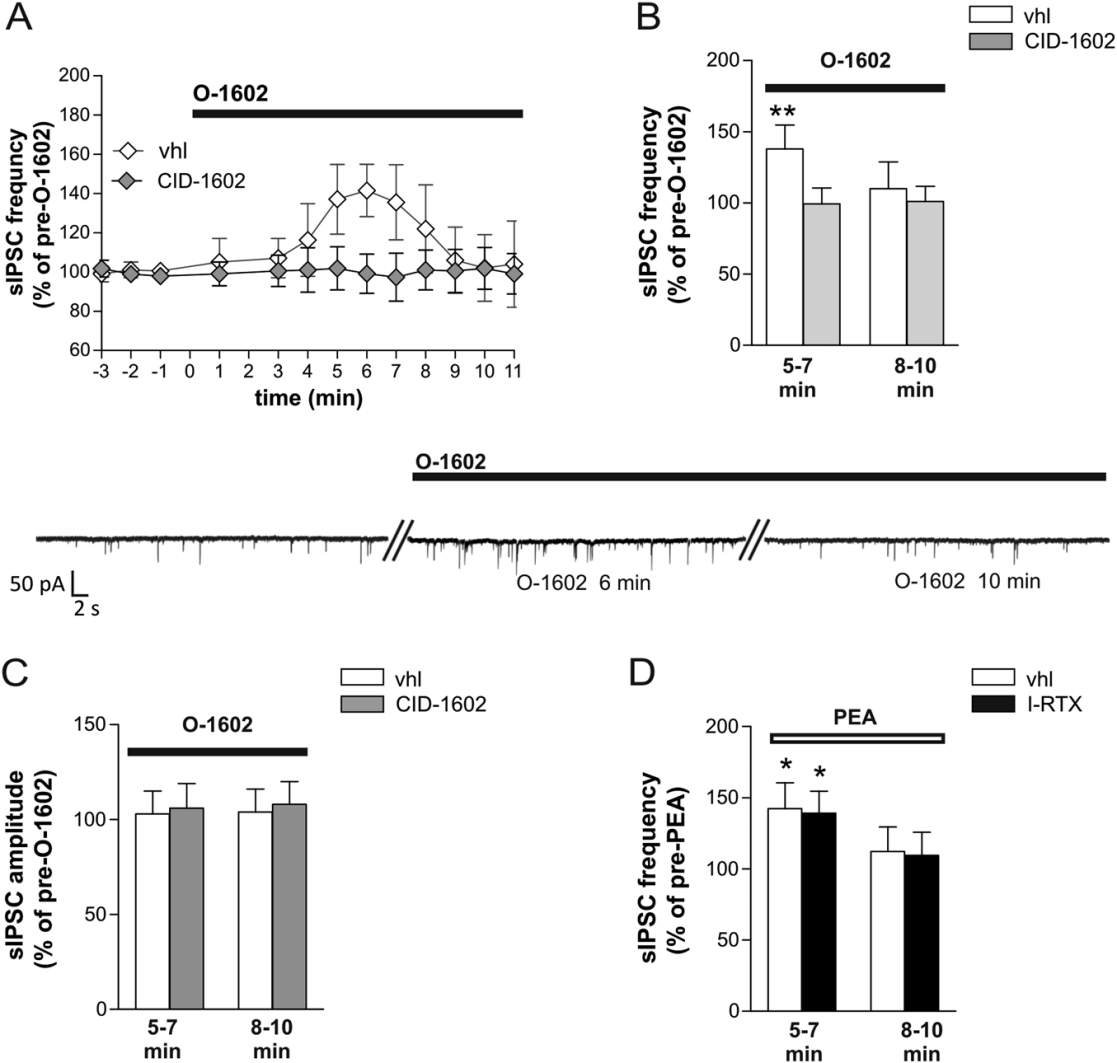
Effect of GPR55 on striatal GABAergic synaptic transmission. (**A,B**) O-1602 caused a rapid and transient increase of sIPSC frequency. Preincubation with CID-1602 prevented the action of O-1602 on sIPSC frequency. The electrophysiological traces on the bottom are examples of voltage-clamp recordings before and during the application of O-1602. (**C**) sIPSC amplitude was unchanged during bath application of O-1602 when applied alone or in the presence of CID-1602. (**D**) Preincubation with the TRPV1 antagonist I-RTX did not affect the PEA-mediated alteration on sIPSC frequency.

Similar transient increase of the frequency of GABA events was recorded after bath application of O-1602, the synthetic agonist of GPR55 receptor and, as expected, it was fully abolished in the presence of CID-1602 (Fig. 2A–C). The electrophysiological results, obtained with either PEA or O-1602, are indicative of a GPR55 presynaptic site of action able to control GABA release.

To further investigate the GPR55-mediated effect on GABA transmission within the striatum, we recorded the effect of PEA on GABA transmission in the presence of TRPV1 antagonist (5′-Iodoresiniferatoxin, I-RTX), since PEA is a weak agonist of TRPV1 channels^3, 21^ Application of I-RTX (1 μM) did not alter the biphasic effect of PEA on inhibitory currents, excluding the involvement of TRPV1 channels on PEA-mediated synaptic effects (Fig. 2D).

### Role of GPR55 on striatal glutamatergic transmission

**Figure 3:**
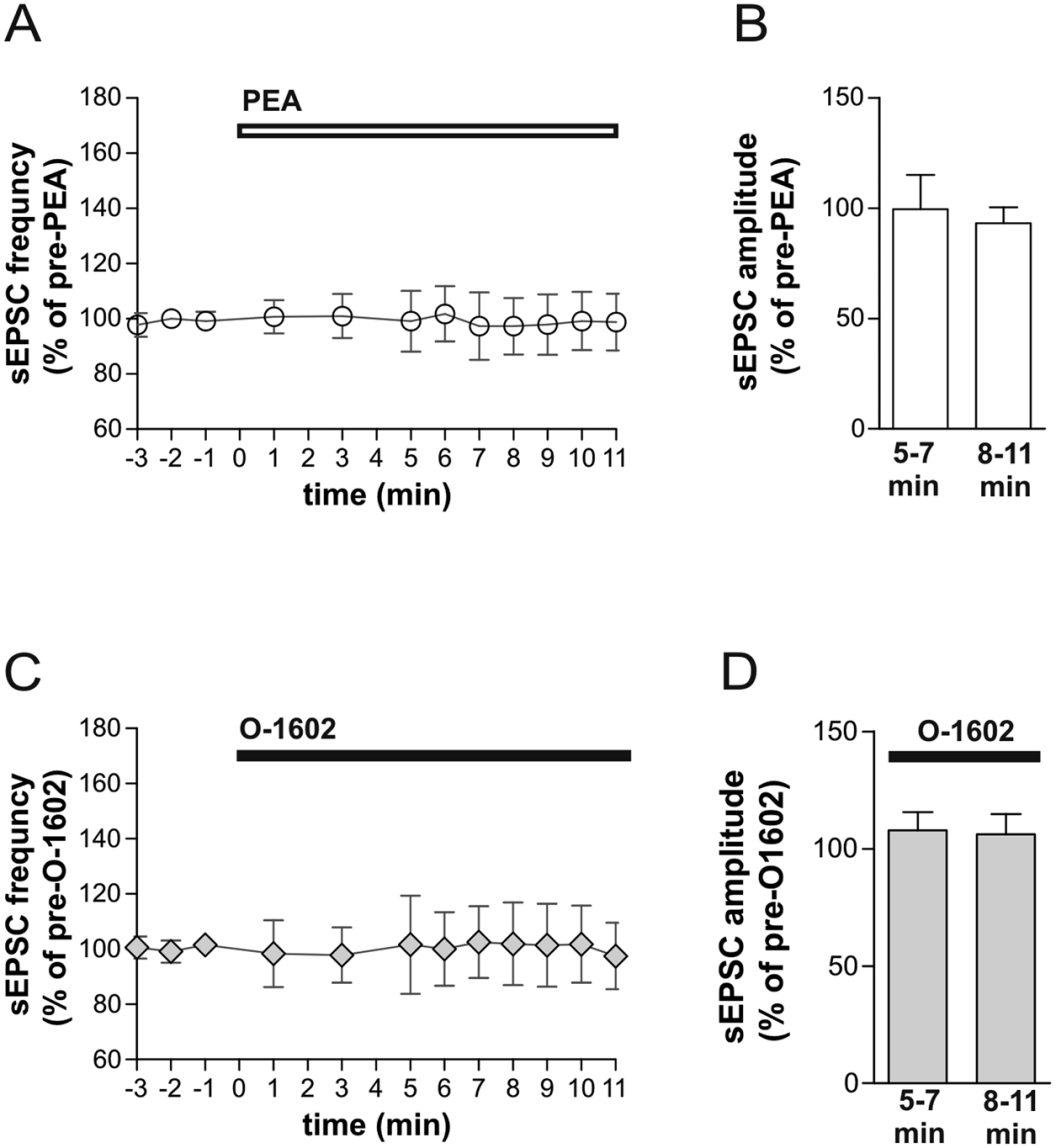
The stimulation of GPR55 receptor fails to alter striatal glutamatergic transmission. (**A,B**) The graphs show that PEA failed to alter the frequency (**A**) and amplitude (**B** *)* of sEPSCs. (**C**,**D**) O-1602 also failed to affect glutamatergic transmission in terms of frequency (**C**) and amplitude (**D**).

In order to further characterize the synaptic effects mediated by PEA and O-1602 within the striatum, we also investigated whether GPR55 receptor agonists are able to affect glutamatergic transmission. Both PEA and O-1602 failed to alter frequency and amplitude of glutamate-mediated spontaneous excitatory postsynaptic currents (sEPSCs) (100% of pre-PEA after 5–7 min; p > 0.05 and 100% of pre-O-1602 after 5–7 min; p > 0.05) (Fig. 3A–D). Application of either PEA or O-1602 also failed to affect sEPSC frequency and amplitude after longer incubations (8–10 min; p > 0,05) (Fig. 3A–D).

### The interplay between GPR55 and CB1 receptors

Within the endocannabinoid system (ECS), cannabinoid type-1 receptor (CB1) is one of the most important regulator of synaptic transmission in striatal neurons^22–24^. Activation of CB1 receptors modulates both excitatory^25–28^ and inhibitory synaptic transmission through a presynaptic action^29^.

**Figure 4:**
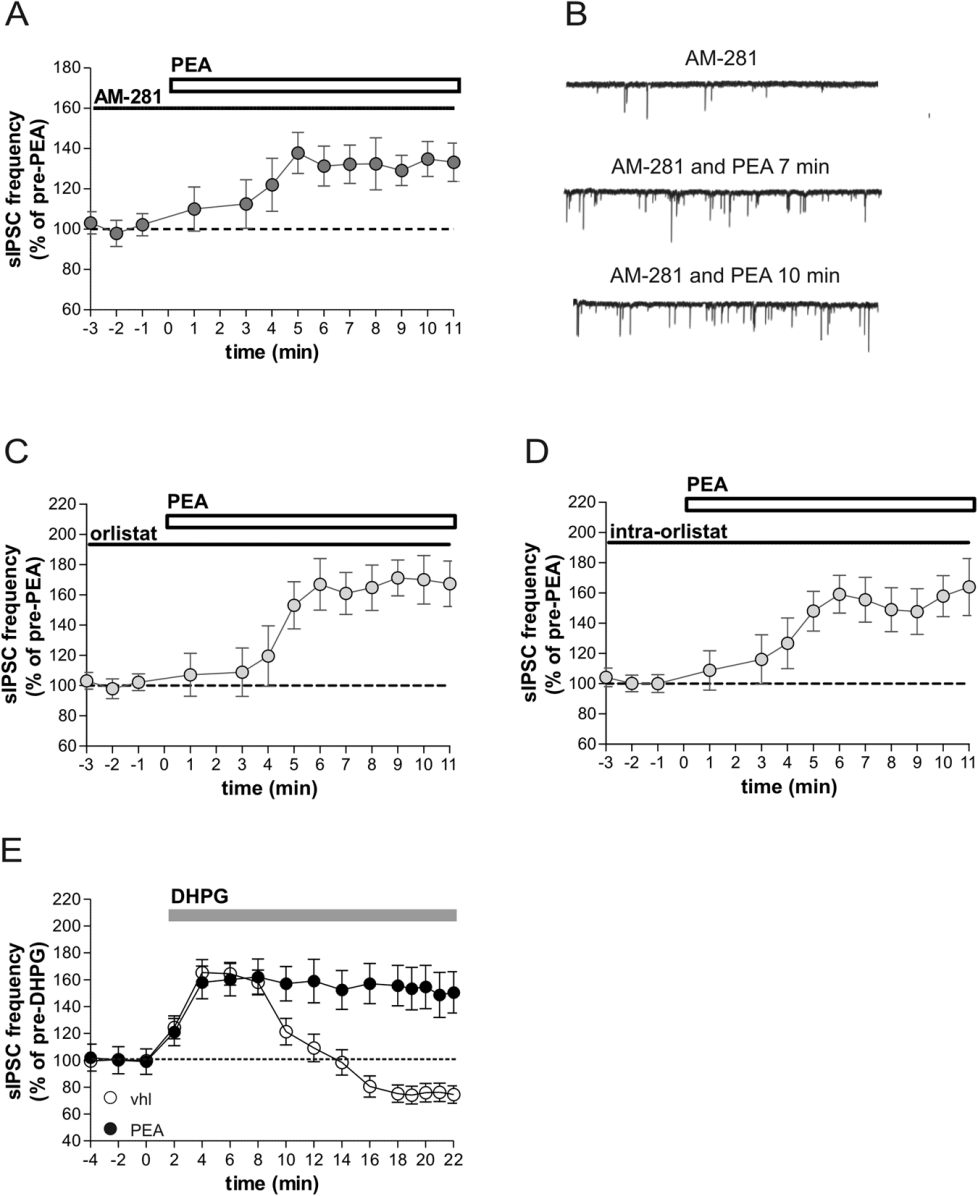
Role of 2AG in the rundown effect observed during PEA incubation. (**A**) The graph shows that preincubation with the CB1 receptor antagonist AM-281 prevented the late depressant action of PEA on sIPSCs. (**B**) Examples of voltage-clamp recordings in the presence of AM281 before and after PEA application. (**C,D**) In these graphs it is shown that the blocker of 2AG synthesis orlistat, either when applied in the external solution (**C**) or when dissolved in the intraelectrode solution (**D**), abolished the rundown effect of PEA on sIPSCs. (**E**) Preincubation with PEA prevented the reduction of sIPSC frequency mediated by 3,5-DHPG.

We tested therefore if CB1 receptors were involved in the synaptic effects of PEA. Bath application of AM281, a selective antagonist of CB1 receptors, did not alter the increase of GABA currents observed after 5–7 minutes after PEA (Fig. 4A and B), confirming the selective role of GPR55 in the modulation of GABA transmission in the striatum. Conversely, sIPSC frequency-time histogram indicated that AM281 was able to prevent the rundown effect observed after 8 min of PEA (Fig. 4A and B), identifying a novel functional interplay between GPR55 and CB1 receptors (n = 7, p < 0.01 at 5–7 min, p < 0.05 compared to pre-drug values at 8–10 min).

The endocannabinoid 2AG is an endogenous agonist of CB1 receptors and acts as “retrograde” neurotransmitter released from somata and/or dendrites of neurons. The release on demand of 2AG from postsynaptic terminals mediates the inhibition of GABA transmission through activation of CB1 receptor on GABAergic axon terminals^29–31^ Consequently, the rundown effect on GABA release observed during PEA incubation could be promoted by a postsynaptic induction of 2-AG synthesis. In order to verify this interplay between PEA and 2AG, electrophysiological recordings were performed incubating PEA in striatal slices treated with orlistat, a lipase inhibitor which blocks the 2-AG synthesis by inhibition of DAG lipase-α1^32^. As reported in Fig. 3C the rundown effect was completely abolished in the presence of orlistat 1 µM (n = 8, p < 0.05 compared to pre-drug values at 5–7 min and 8–10 min), suggesting an interaction between PEA and 2-AG synthesis.

To confirm that PEA was able to induce synthesis and synaptic effects of 2-AG by acting postsynaptically, we also tested the effects of intraneuronal delivery of orlistat, by dissolving this DAG-lipase inhibitor in the intraelectrode solution. As reported for the experiment performed by dissolving orlistat in the external solution, the DAG-lipase inhibitor fully prevented the rundown effect on sIPSC frequency observed after 8–10 min of PEA (n = 6, p < 0.05 compared to pre-drug values at 5–7 and 8–10 min) (Fig. 4D), confirming an action of PEA on postsynaptic cell.

Previous studies have reported that activation of metabotropic glutamate receptor 5 (mGluR5) was able to modulate GABA transmission in the striatum, causing a biphasic effect on sIPSC frequency reminiscent of the effects of PEA. 3,5-DHPG, an agonist of group I mGlu receptors, in fact, initially increased and then reduced the frequency of these synaptic events^33^. Interestingly, the latest effect of 3,5-DHPG was mediated by a postsynaptic release of 2AG promoted by the enhancement of its synthesis, which in turn stimulated CB1Rs on GABAergic nerve terminals and depressed the frequency of inhibitory currents in the striatum^33, 34^. Thus, to further see whether PEA also interfered with the synthesis of this endocannabinoid and consequently with the stimulation of CB1 receptors, we investigated whether the effect of 3,5-DHPG and that of PEA were occlusive. In accordance with previous findings, 3,5-DHPG showed a late depressant action on GABA transmission reducing the frequency of sIPSCs in striatal slices (at 20 min, 83 ± 6,5%, p < 0.05), an effect that was ascribed to stimulation of 2AG synthesis and that was absent in PEA bathed slices (sIPSC frequency in PEA plus 3,5-DHPG at 20 min, 168 ± 10,1%; n = 5, p > 0.05) (Fig. 4E). These data suggest that mGluR5 and GPR55 mediate stimulation of CB1 receptors through similar mechanisms depending on stimulation of 2AG synthesis.

## Discussion

In this electrophysiological study, we have identified a previously unrecognized function of PEA, demonstrating that GABAergic transmission is under the control of this compound in the striatum.

Our data show that stimulation of GPR55 receptor by endogenous (PEA) or synthetic (O-1602) congeners of anandamide transiently increases GABAergic, but not glutamatergic, transmission recorded from striatal neurons. Moreover, the results of the present study extend the previously described ‘entourage’ effect of PEA on 2-AG, indicating that PEA modulates the release of the endocannabinoid 2-AG.

Several pathways have been suggested to mediate the effects of PEA in the CNS^9, 35^ among which the interaction with GPR55 is widely recognized. In the human brain, qRT-PCR studies have found that highest levels of GPR55 were detected in the basal ganglia, with lowest expression levels in the cerebellum^36^. A similar distribution was reported in mouse brain^15^.

The results of the present study demonstrate a direct role of GPR55 in the neuromodulatory action mediated by PEA in the striatum. The selective effects of PEA on GABA transmission were fully prevented by pharmacological inactivation of GPR55 receptors and were completely replicated by the synthetic agonist of these receptors, O-1602. Moreover, although a weak TRPV1 channel affinity for PEA has been described^3, 21^, the results of the present study argue against a direct pharmacological action of PEA on these channels, since we have found that the effects of PEA on sIPSC frequency were fully preserved in the presence of I-RTX, a TRPV1 antagonist. Such results indicated that the PEA-mediated increase of GABA currents in medium spiny neurons was entirely mediated by GPR55.

A presynaptic action was likely responsible for the effects of GPR55 stimulation, since the effects of PEA or O-1602 on sIPSC frequency were not associated with significant changes of sIPSC amplitude, which largely depends on the postsynaptic properties of the recorded neurons. Activation of GPR55 has been associated with transiently increased Ca^2+^ release from presynaptic Ca2+ stores in hippocampal slices and in dorsal root ganglion neurons^16, 37^, an effect which might account for the observed increase of striatal GABA release by PEA and O1602 in striatal neurons. It should be noted, however, that in hippocampus such effect was associated to an increase of excitatory frequencies in CA3-CA1 synapses^37^. Therefore, it seems likely that the mechanism described herein applies only in selected brain areas and further investigations are required to understand the mechanism through which PEA produces this synaptic effect.

In the striatum, GPR55 co-localizes with cannabinoid CB1R^9, 38^, suggesting a close functional interaction between these distinct cannabinoid receptors. Several studies have demonstrated that the release on demand of 2-AG is an important mediator of inhibitory transmission by activation of pre-synaptic CB1R located on GABA nerve terminals within the striatum^33, 34, 39^. We studied PEA-mediated modulation of 2-AG metabolism, and our results show that the delayed decrease of GABA inputs after PEA stimulation is a result of its action on GPR55 on postsynaptic site, and is mediated by the endocannabinoid 2-AG through the stimulation of CB1R. Pharmacological inhibition of 2-AG synthesis by orlistat was in fact able to abrogate the rundown effect observed during PEA incubation. Moreover, since mGlu5 receptors have been previously found to reduce inhibitory currents by post-synaptic release of 2-AG^33, 34^, the occlusive effect on sIPSC frequency promoted by concomitant application of PEA and mGlu5 receptor agonist (3,5-DHPG), suggests that PEA may induce 2AG synthesis, thus sharing a common pathway with 3,5-DHPG. In line with this, a recent biochemical study showed that PEA exerts significant effect on endocannabinoid 2-AG by enhancing both its levels in keratinocytes and in human and canine plasma^21, 40^. Of note, since PEA exerts profound anti-inflammatory effects^41, 42^ and pro-inflammatory cytokines are able to affect synaptic transmission^43–46^, an indirect effect of PEA, through the modulation of cytokines, might contribute to GABA transmission alteration. In conclusion, our electrophysiological study identifies a novel synaptic function of PEA, and extends our knowledge on how the endocannabinoid system controls synaptic transmission and controls itself.

## Methods

### Animals

The subjects in this study were 7–9 weeks old female mice, C57BL/6 N, obtained from Charles-River (Italy) and CNR-EMMA Mouse Clinic facility (Monterotondo-Rome, Italy). Animals were randomly assigned to standard cages, with four to five animals per cage, and kept at standard housing conditions with a light/dark cycle of 12 h and free access to food and water. Animal experiments were carried out according to the Internal Institutional Review Committee, the European Directive 2010/63/EU and the European Recommendations 526/2007 and the Italian D.Lgs 26/2014. The experimental protocol was approved by Italian Ministry of Health (protocol number 35/2014B).

### Electrophysiology

Mice were killed by cervical dislocation, and corticostriatal coronal slices (190 µm) were prepared from fresh tissue blocks of the brain with the use of a vibratome^47, 48^. A single slice was transferred to a recording chamber and submerged in a continuously flowing artificial CSF (ACSF) (34 °C, 2–3 ml/min) gassed with 95% O2–5% CO2. The composition of the control ACSF was (in mM): 126 NaCl, 2.5 KCl, 1.2 MgCl2, 1.2 NaH2PO4, 2.4 CaCl2, 11 Glucose, 25 NaHCO3. The striatum could be readily identified under low power magnification, whereas individual neurons were visualized *in situ* using a differential interference contrast (Nomarski) optical system. This employed an Olympus BX50WI (Japan) non-inverted microscope with 40x water immersion objective combined with an infra-red filter, a monochrome CCD camera (COHU 4912), and a PC compatible system for analysis of images and contrast enhancement (WinVision 2000, Delta Sistem, Italy). Recording pipettes were advanced towards individual striatal cells in the slice under positive pressure and visual control (WinVision 2000, Delta Sistemi, Italy) and, on contact, tight GΩ seals were made by applying negative pressure. The membrane patch was then ruptured by suction and membrane current and potential monitored using Multiclamp700B and Digidata 1440 A by Axon Instrument (Molecular Devices, Foster City, CA, USA). Whole-cell access resistances measured in voltage clamp were in the range of 5–20 MΩ. Whole-cell patch clamp recordings were made with borosilicate glass pipettes (1.8 mm o.d.; 2–3 MΩ), in voltage-clamp mode, at the holding potential (HP) of −80 mV.

To study GABA-mediated spontaneous inhibitory postsynaptic currents (sIPSCs), the recording pipettes were filled with internal solution of the following composition (mM): 110 CsCl, 30 K+−gluconate, 1.1 EGTA, 10 HEPES, 0.1 CaCl2, 4 Mg-ATP, 0.3 Na-GTP. MK-801 and CNQX were added to the external solution to block, respectively, NMDA and non-NMDA glutamate receptors.

To detect glutamate-mediated spontaneous excitatory postsynaptic currents (sEPSCs), the recording pipettes were filled with internal solution of the following composition (mM): K+−gluconate (125), NaCl (10), CaCl2, (1.0), MgCl2 (2.0), 1,2-bis (2-aminophenoxy) ethane-N,N,N,N-tetraacetic acid (BAPTA; 0.5), N-(2-hydroxyethyl)-piperazine-N-s-ethanesulfonic acid (HEPES; 19), guanosine triphosphate (GTP; 0.3), Mg-adenosine triphosphate (Mg-ATP; 1.0), adjusted to pH 7.3 with KOH. Bicuculline (10 µM) was added to the perfusing solution to block GABAA-mediated transmission.

Synaptic events were stored by using P-CLAMP 10.6 (Axon Instruments) and analyzed off line on a personal computer with Mini Analysis 6.0.7 (Synaptosoft, Leonia, NJ, USA) software. The detection threshold of spontaneous excitatory and inhibitory events was set at twice the baseline noise. The fact that no false events would be identified was confirmed by visual inspection for each experiment. Offline analysis was performed on spontaneous synaptic events recorded during fixed time epochs (1 min). Only cells that exhibited stable frequencies in control (less than 20% changes during the control samplings) were taken into account.

Only data from putative GABAergic medium spiny projection neurons (MSNs) were included in the present study and identified immediately after rupture of the GΩ seal, by evaluating their firing response to the injecting of depolarizing current (typically tonic, with little or no adaptation).

One to five cells per animal were recorded. For each type of experiment and time-point, at least 3 mice per group were employed. One animal per day was used for the electrophysiological experiment. Throughout the text “n” refers to the number of cells, unless otherwise specified.

### Drugs

Drugs used for the electrophysiological experiments were applied by dissolving them to the desired final concentration in the bathing ACSF. The concentrations of the various drugs were as follows: AM281 (2 μM), 5′-Iodoresiniferatoxin (I-RTX, 1 μM), CNQX (10 μM), MK-801 (25 μM), orlistat (5 μM), N-palmitoylethanolamine (PEA, 10 μM), O-1602 (10 μM), CID-16020046 (CID-1602, 20 μM), (S)-3,5-DHPG (50 μM) (from Tocris, Bristol, UK). Bicuculline (10 μM) (from Sigma-RBI, St. Louis, USA). Bicuculline, CNQX, (S)-3,5-DHPG and MK-801 were dissolved in water. AM281, I-RTX, orlistat, O-1602 and CID-16020046 were dissolved in DMSO. PEA was dissolved in ethanol. The intraneuronal delivery of orlistat was performed dissolving the drug (orlistat, 5 µM) in the internal solution.

### Statistical analysis

Data were presented as mean ± SEM. Throughout the text “n” refers to the number of cell recorded. A paired Student’s t-Tests was used to compare two population means, before and after drug incubations on the same cells. The significance level was established at p < 0.05.
